# Orbital and eyelid diseases: The next breakthrough in artificial intelligence?

**DOI:** 10.3389/fcell.2022.1069248

**Published:** 2022-11-18

**Authors:** Xiao-Li Bao, Ying-Jian Sun, Xi Zhan, Guang-Yu Li

**Affiliations:** ^1^ Department of Ophthalmology, Second Hospital of Jilin University, Changchun, China; ^2^ Department of Engineering, The Army Engineering University of PLA, Nanjing, China; ^3^ The Eye Hospital, School of Ophthalmology & Optometry, Wenzhou Medical University, Wenzhou, China

**Keywords:** artificial intelligence, deep learning, orbital and eyelid diseases, ophthalmic plastic surgery, orbital computed tomography, orbital magnetic resonance imaging

## Abstract

Orbital and eyelid disorders affect normal visual functions and facial appearance, and precise oculoplastic and reconstructive surgeries are crucial. Artificial intelligence (AI) network models exhibit a remarkable ability to analyze large sets of medical images to locate lesions. Currently, AI-based technology can automatically diagnose and grade orbital and eyelid diseases, such as thyroid-associated ophthalmopathy (TAO), as well as measure eyelid morphological parameters based on external ocular photographs to assist surgical strategies. The various types of imaging data for orbital and eyelid diseases provide a large amount of training data for network models, which might be the next breakthrough in AI-related research. This paper retrospectively summarizes different imaging data aspects addressed in AI-related research on orbital and eyelid diseases, and discusses the advantages and limitations of this research field.

## Introduction

Artificial Intelligence (AI) simulates and extends human intelligence, and has been hailed as “the Future of Employment” (Yang et al., 2021). Long before the mid-twentieth century, the British scientist Alan Turing first predicted that machines could become intelligent (Li et al., 2019), and in 1956, McCarthy introduced “AI” at the Dartmouth Conference (Dzobo et al., 2020). At the time, “AI” was actualized *via* a static computer program that controlled a machine, which is unlike the AI we currently know (Mintz and Brodie, 2019). In 1959, Samuel developed the theory of AI and proposed “machine learning (ML)” (Finlayson et al., 2019), which denotes the capability of a computer to learn by itself without explicit program instructions (Nichols et al., 2019). In ML large amounts of data are analyzed to make predictions on real-world events using supervised and unsupervised algorithms. ML has spawned variants such as conventional machine learning (CML) and deep learning (DL) (Brehar et al., 2020; Ye et al., 2020). DL has exhibited a remarkable ability to analyze high-dimensional data with multiple processing layers, gradually becoming the mainstream of ML modeling (Finlayson et al., 2019). In particular, DL-based technologies display excellent abilities to extract image features and associate various types of data, which plays an active role in the automatic recognition of image, sound, and text data (Cai et al., 2020). Ting et al. (2017) reported that AI could automatically diagnose diabetic retinopathy from more than 100,000 retinal photographs. In recent years, DL has gradually become a new tool in the automatic diagnosis of glaucoma and cataracts (Lin et al., 2019; Wu et al., 2019; Wang et al., 2020a; Girard and Schmetterer, 2020). Some commercial software applications related to DL are used to assist in the diagnosis of retinal diseases in clinical practice (van der Heijden et al., 2018; Girard and Schmetterer, 2020).

Imaging data, including orbital computed tomography (CT), orbital magnetic resonance imaging (MRI), and external ocular photographs, play a crucial role in the diagnosis and treatment of orbital and eyelid diseases (Bailey and Robinson, 2007; Abdullah et al., 2010). Currently, AI automatically diagnoses and grades some orbital and eyelid diseases, such as orbital blowout fractures and thyroid-associated ophthalmopathy (TAO) (Li et al., 2020; Song et al., 2021a). Automatic measurement of eyelid morphological parameters and automatic surgical decision-making based on AI technology are two recent research hotspots (Bahceci Simsek and Sirolu, 2021; Chen et al., 2021; Lou et al., 2021; Hung et al., 2022). Compared to traditional medical models, AI can rapidly analyze large sets of patient data, achieving healthcare cost savings and assisting in the construction of teleconsultation platforms (Bi et al., 2020). Automatic measurement of eyelid morphological parameters based on AI technology could correct artifactual errors to maintain objectivity and repeatability in patient data evaluation, which might be a new tool in the assessment of oculoplastic surgery (Lou et al., 2021). However, because of the small amount of standard imaging data and the imbalance in categories, ensuring a highly-efficient algorithm training is still a challenge. In addition, the development of methods for obtaining high-quality imaging data of orbital and eyelid diseases should also be considered.

In this paper, we comprehensively review the application of AI-based technology to the diagnosis and treatment of orbital and eyelid diseases by analyzing various types of image data. The advantages and limitations of AI in this field are also discussed to explore its potential targets in detecting and treating orbital and eyelid diseases.

### What is artificial intelligence?

AI is a branch of computer science, in which “artificial” indicates that the systems are man-made and “intelligence” denotes features such as consciousness and thinking (Thrall et al., 2018). The major purpose of AI is to simulate human thinking processes by learning from existing experiences to solve problems that cannot be solved through traditional computer programming (Bischoff et al., 2019). ML is a subset of AI that has become the mainstream of AI technology (Shin et al., 2021). ML extracts and analyzes the features of input samples to classify new homogeneous samples (Totschnig, 2020). ML automatically improves and optimizes computer algorithms and programs by analyzing the data rather than relying on explicit program instructions (Nichols et al., 2019; Cho et al., 2021). Among the various ML models that have emerged, neural networks simulate the synaptic structure of human neurons and improve the computational ability of ML by adjusting the parameters of network models (Starke et al., 2021). Convolutional neural networks (CNNs), which have an encoding structure similar to that of visual cortical neurons (Hou et al., 2019), have become one of the most popular neural network models (Mintz and Brodie, 2019).

**FIGURE 1:**
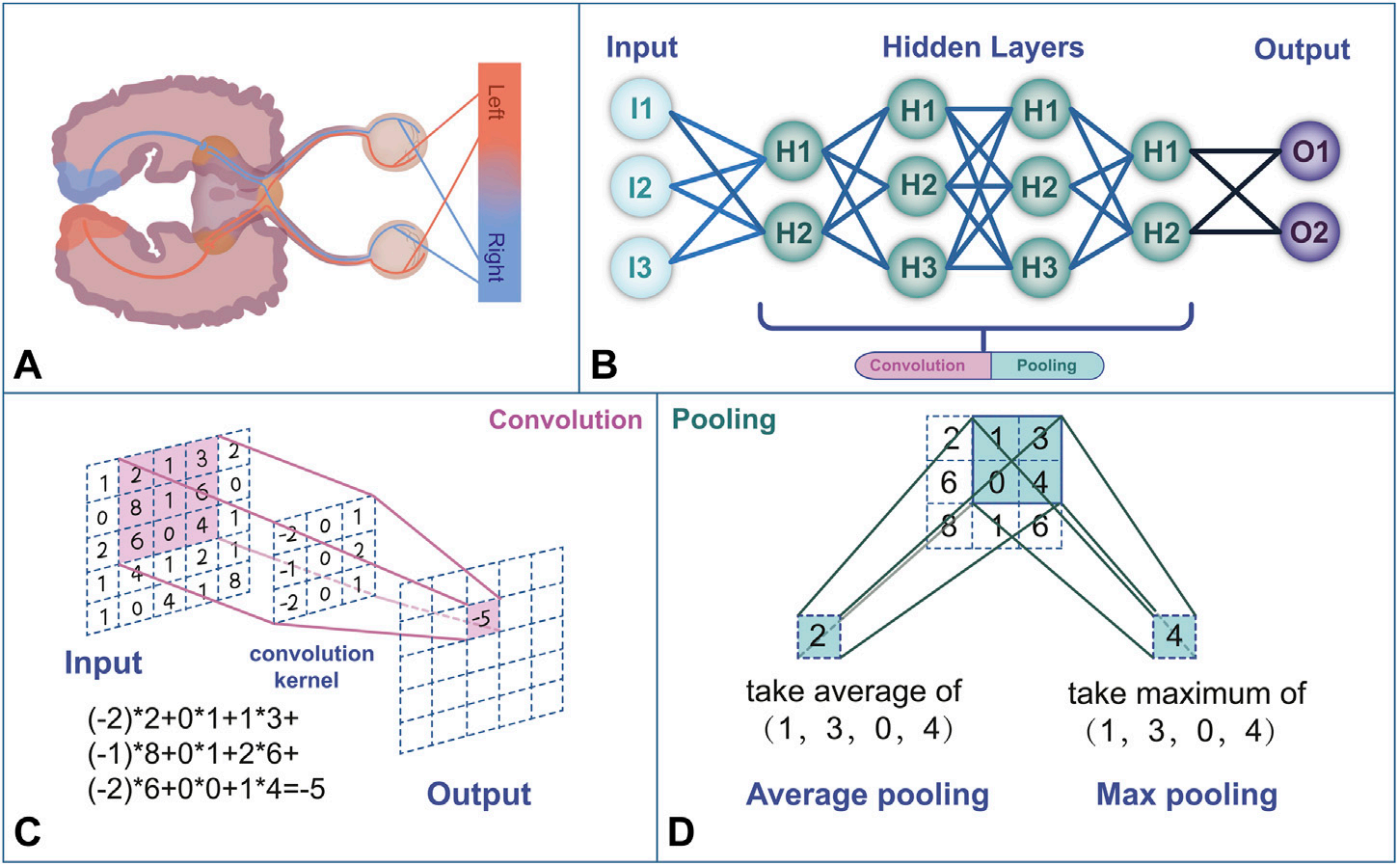
**(A)** Human visual feedback pathway. **(B)** Neural network structure framework mimics the human neural network. **(C)** The convolution process performs a linear transformation at each position of the image and maps it to a new value. **(D)** Pooling is a computational process that reduces the data size. The commonly used pooling methods are max pooling and average pooling.

In human vision, each neuron in the visual cortex responds to stimulation by activating specific regions in the visual space that form the entire visual field (Figure 1) (Brachmann et al., 2017). Similarly, CNNs extract features from the input image and output a feature map using convolution and pooling operations (Le et al., 2020). A convolution layer consists of a set of two-dimensional numerical matrices that are also known as filters. The CNN obtains the pixel value of the output images by multiplying the value in the filter by the value of the corresponding pixel in the image and summing the product, that is, *via* convolution operations (Brachmann et al., 2017). To avoid similar sizes of the output pixels after the convolution operation, the CNN changes the size of the output pixels by reducing the input values through the pooling operation. By repeating the convolution and pooling operations, the CNN continuously self-corrects so that the output values become closer to the human ratings (Larentzakis and Lygeros, 2021). New neural network models, such as UNet and ResNet, have been developed to overcome the difficulty of training CNNs with deep layers. These neural network models improve the framework of a CNN by expanding its depth, convolutional layer, or pooling layer. For example, while traditional CNN models can only classify images and output the labeling of an entire image, UNet can achieve pixel-level classification and output the class of each pixel, which makes it well-suited for image segmentation tasks (Yin et al., 2022). ResNet solves the gradient vanishing and gradient exploding problems of traditional CNNs by adding a residual block (He et al., 2020).

**FIGURE 2:**
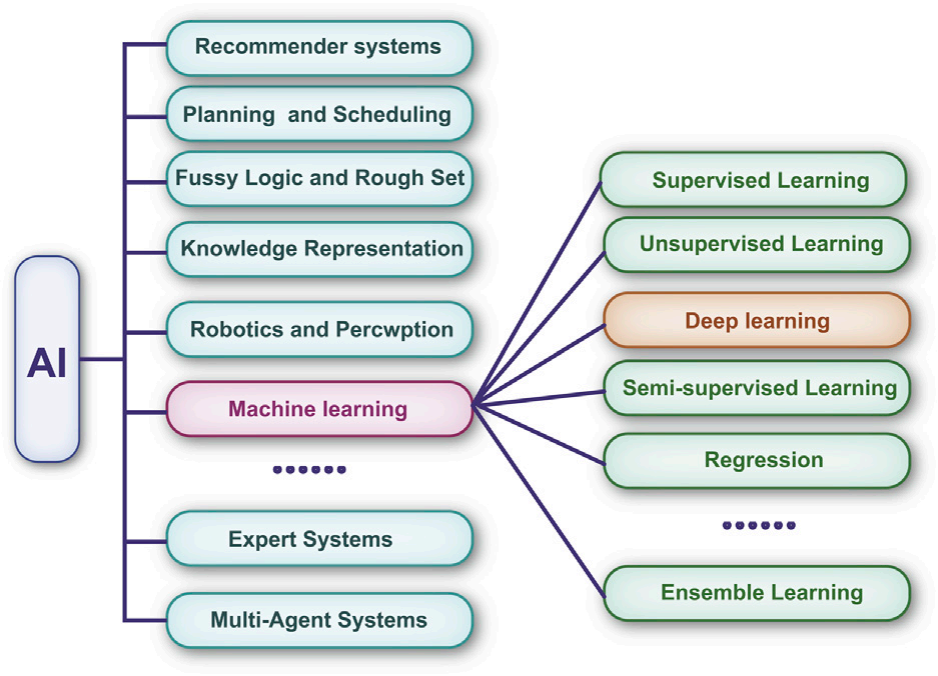
Machine learning is a subset of artificial intelligence. Deep learning has revolutionized the machine learning field in the past few years. It is now widely used in image recognition, voice recognition, etc.

To process large amounts of data, multilayer neural networks have been cascaded to form DL algorithms (Kaluarachchi et al., 2021). Compared with traditional ML algorithms, DL has a greater ability to analyze large-scale matrix data (Jalali et al., 2021). The relationship between AI, ML, and DL is shown in Figure 2. Currently, DL-based technologies are widely used in the diagnosis of certain ophthalmic diseases, such as cataracts (Wu et al., 2019) and glaucoma (Sudhan et al., 2022), and the segmentation of medical images, including those of retinal vessels (van der Heijden et al., 2018).

## Artificial intelligence technology applied to orbital computed tomography/magnetic resonance imaging images

Orbital CT and MRI are important tools for the diagnosis and monitoring of orbital and eyelid diseases (Weber and Sabates, 1996). MRI and CT rely on magnetic fields and radio wave energy to provide images within the orbit (Russell et al., 1985; Langer et al., 1987). MRI is suitable for imaging soft tissue, whereas CT is commonly used to image bony structures (Hou et al., 2019). CT and MRI images are suitable as training data for AI-based research as they have less background occupation and noise (Thrall et al., 2018; Jalali et al., 2021).

### Automatic identification and segmentation of anatomical structures from orbital computed tomography/magnetic resonance imaging

Automatic recognition and labeling of anatomical structures of the eye orbit can be achieved through the segmentation of medical images based on AI technology (Hou et al., 2019). Furthermore, AI can segment bony structures from orbital CT/MRI images. Hamwood et al. (2021) developed a DL system for the segmentation of bony regions from orbital CT/MRI images that exhibited excellent efficiency, particularly in terms of computational time. Li et al. (2022a) extracted bony orbit features and analyzed Asian aging characteristics through the popular deep CNN (DCNN) model. Some commercial software can also automatically segment orbital regions from CT images (Hamwood et al., 2021). In addition, using AI-based technology, irregular soft tissues, such as fat and abscesses, have been reliably segmented from orbital CT/MRI images. Brown et al. (2020) used a UNet-like CNN to segment orbital septal fat from orbital MRI images, and the results showed that AI segmentation was consistent with manual segmentation. Fu et al. (2021) trained and evaluated a context-aware CNN (CA-CNN) to segment orbital abscess regions from CT images of patients with orbital cellulitis, with the AI results being similar to those obtained by medical experts.

In addition, AI can automatically quantify certain anatomical structures based on image segmentation. Umapathy et al. (2020) established an MRes-UNet model to segment and quantify the volume of the eyeball based on orbital CT images. Pan et al. (2022) achieved automatic calculation of the size and height of the bony orbit regions using a U-Net++ based on pre-3D images reconstructed from orbital CT images.

### Automatic diagnosis and grading of orbital and eyelid diseases based on orbital computed tomography/magnetic resonance imaging images

Orbital CT/MRI images are crucial in the preliminary diagnosis of orbital diseases such as orbital wall fractures, orbital tumors, and TAO (Griffin et al., 2018). Orbital blowout fractures are one of the most common injuries caused by orbital trauma. Li et al. (2020) used the Inception V3 DCNN to automatically classify CT images exhibiting orbital burst fractures. Song et al. (2021a) proposed a 3D-ResNet to automatically detect TAO from orbital CT images, and the trained AI algorithm showed excellent performance in a real clinical setting. Lin et al. (2021) used a DCNN to grade TAO based on orbital MRI images, resulting in a labeling of disorder areas that was consistent with that made manually through an occlusion test. Hanai et al. (2022) developed a deep neural network to assess the enlarged extraocular muscles (EEM) of patients with Graves’ ophthalmopathy (GO) from orbital CT images. When applied to the test data, the area under the receiver operating curve (AUC) was 0.946, indicating that the deep neural network could effectively detect EEM in GO patients. Lee et al. (2022) used 288 orbital CT scans from patients with mild and moderate-to-severe GO and healthy controls to train a neural network for diagnosing and assessing the severity of GO. The developed neural network yielded an AUC of 0.979 in diagnosing patients with moderate-to-severe GO. Han et al. (2022) automatically identified the differences in the orbital cavernous venous malformations (OCVM) from orbital CT images by training 13 ML models, including support vector machines (SVMs) and random forests. Nakagawa et al. (2022) implemented a VGG-16 network to determine from CT images whether a nasal or sinus tumor invades the periorbital area. The network model achieved a diagnostic accuracy of 0.920, indicating that CNN-based DL techniques can be a useful supporting tool for assessing the presence of orbital infiltration on CT images.

In addition to diagnosing and grading diseases, AI can extract and determine subtle features from images to differentiate confusing diseases. Orbital cavernous hemangioma and schwannoma differ in terms of surgical strategy but have similar MRI features. Bi et al. (2020) developed a database of orbital MRI images of patients with cavernous hemangioma and schwannoma from 45 hospitals in China and used AI to identify and classify the affected eye, tumor location, and tumor category. The AI system was validated, showing an accuracy greater than 0.900 on a multicenter database. Xie et al. (2022) developed a DL model that combines multimodal radiomics with clinical and imaging features to distinguish ocular adnexal lymphoma (OAL) from idiopathic orbital inflammation (IOI). The diagnosing results yielded an AUC of 0.953, indicating that the DL-based analysis may successfully help distinguish between OAL and IOI. Hou et al. (2021) used an SVM classifier and the bag-of-features (BOF) technique to distinguish OAL from IOI based on orbital MRI images. During an independent verification test, the proposed method with augmentation achieved an AUC of 0.803, indicating that BOF-based radiomics might be a new tool for the differentiation between OAL and IOI. Early detection of hypothyroid optic neuropathy (TON) is crucial in clinical decision-making. Wu et al. (2022) built an AI predictive model to distinguish between TAO and TON by extracting radiomic features from optic-nerve T2-weighted water-fat images from a cohort of patients with TAO and a cohort of patients with TON. Table 1 summarizes the discussed AI-related studies on orbital CT/MRI images.

**TABLE 1:** AI-related studies utilizing orbital CT/MRI images.

Authors	Study goals	Imaging data type	Dataset	Network model	Accuracy	AUC	Dice
Hamwood et al. (2021)	Segmentation of the bony orbital regions	Orbital CT and MRI images	Training set (*n* = 443 slices). Test set (*n* = 363 slices)	Two full convolutional neural networks (CNNs) in series followed by a graph-search method	—	—	Dice (CT images) = 0.813 and 0.975. Dice (MRI images) = 0.930 and 0.995
Li et al. (2022a)	Analysis of Asian aging characteristics by extracting features of the bony orbit	Orbital CT images	595 people	UNet	0.979 (Male) and 0.992 (Female)	—	—
Brown et al. (2020)	Segmentation of orbital septal fat	Orbital MRI images	1,018 scans from 256 participants	UNet-like CNN	—	—	—
Fu et al. (2021)	Segmentation of orbital abscess regions	Orbital CT images	67 patients	Context-aware CNN (CA-CNN)	—	—	Dice = 0.780. Jaccard = 0.120. Hausdorff = 0.650
Umapathy et al. (2020)	Segmentation and quantification of eyeball volume	Orbital CT images	80 patients	MRes-UNet	—	—	Dice = 0.940
Pan et al. (2022)	Segmentation of the bony orbit regions	Orbital CT images	595 Chinese people	UNet	—	—	IoU = 0.954
Li et al. (2020)	Classification of orbital CT images with orbital blowout fractures	Orbital CT images	94 patients and 94 normal people	Inception V3 deep CNN (DCNN)	0.920	0.957	—
Song et al. (2021a)	Detection of patients with thyroid-associated ophthalmopathy (TAO)	Orbital CT images	193 patients and 715 normal people	3D-ResNet	0.870	0.919	—
Lin et al. (2021)	Grading of TAO disease	Orbital MRI images	160 patients (80% for training, 20% for testing)	DCNN	0.863	0.922	—
Hanai et al. (2022)	Assessment of the enlarged extraocular muscles of patients with Graves’ ophthalmopathy	Orbital CT images	371 participants		—	0.946	—
Lee et al. (2022)	Diagnosis and severity assessment of Graves’ ophthalmopathy	Orbital CT images	288 cases; 80% for training and 20% for testing	A developed CNN.	Moderate-to-severe GO: 0.930 mild GO: 0.826	0.979 0.895	—
Han et al. (2022)	Distinguishing orbital cavernous venous malformations	Orbital CT images	215 patients with OCVM and 96 non-OCVM patients	13 ML models	—	—	—
Nakagawa et al. (2022)	Determination of whether a tumor invades the periorbital area in a nasal or sinus tumor	Orbital CT images	Training set (*n* = 119). Test set (*n* = 49)	Pre-trained CNN algorithm devoted to image classification	0.920	0.940	—
Bi et al. (2020)	Identification and classification of the affected eye, tumor location, and tumor category	Orbital MRI images	11,489 images of cavernous hemangioma and 3,478 images of schwannoma	RCNN ResNet-101	0.911	0.954	—
Xie et al. (2022)	Distinguishing ocular adnexal lymphoma (OAL) from idiopathic orbital inflammation (IOI)	Orbital CT images	OAL (*n* = 39) and IOI (*n* = 50)	VGG-16	0.920	0.953	—
Hou et al. (2021)	DifferentiatingOAL and IOI	Orbital contrast-enhanced MRI (CE-MRI)	IOI (*n* = 28 patients) and OAL (*n* = 28 patients)	Support vector machine (SVM)	—	0.803	—
Wu et al. (2022)	Distinguishing hypothyroid optic neuropathy from TAO patients	Orbital MRI images (optic-nerve T2-weighted water-fat images)	Training set (*n* = 163). Test set (*n* = 72)	Radiomics nomogram	—	Test set: 0.880 vs. 0.750	—

## Artificial intelligence technology based on external ocular photographs

Owing to features such as easy and convenient delivery and storage, external ocular photographs are unique imaging data for diagnosing orbital and eyelid diseases. External ocular photographs show abnormalities and deformities in the orbital and eyelid appearance caused by trauma, tumors, inflammation, and other factors (Fukuda et al., 2005). With the development of face recognition technology, AI could locate and extract ocular information from faces, which lays the foundation for AI research based on external ocular photographs.

### Automatic measurements of eyelid morphologic parameters from external ocular photographs

The accurate measurement of eyelid morphological parameters is crucial in developing an individual eyelid surgery strategy. However, manual measurement of eyelid morphological parameters is difficult to replicate because of subjective errors induced by head movements and changes in facial expressions. AI provides a more objective and convenient tool for quantifying eyelid morphological parameters by parameterizing facial structures and automatically measuring length, area, and volume. Moriyama et al. (2006) achieved eye motion tracking based on the eyelid structure parameters and iris position. Van Brummen et al. (2021) utilized a ResNet-50 model to segment regions, such as the iris and eyebrow, to measure the marginal reflex distance (MRD) in static and dynamic external ocular photographs. Simsek and Sirolu used computer vision algorithms to automatically measure pupillary distance (PD), eye area (EA), and average eyebrow height (AEBH) from external ocular photographs for evaluating the surgical effect of patients who had undergone Muller’s muscle-conjunctival resection (MMCR) surgery (Bahceci Simsek and Sirolu, 2021). The automated measurement of eyelid morphology parameters based on AI technology helps assess eyelid status and improves the accuracy of eyelid surgery.

Compared with other types of imaging data, external ocular photographs can be taken and shared by patients and physicians through smartphones and the internet, which provides sufficient data for AI research on external ocular photographs. Chen et al. (2021) compiled CNN algorithms using the software MAIA to build DL models for the automatic measurement of MRD1, MRD2, and levator muscle strength based on external ocular photographs taken with smartphones. This study was the first smartphone-based DL model for the automatic measurement of eyelid morphological parameters. Compared to those obtained manually, measurements taken with the aid of AI are more objective.

Ptosis is a common eyelid disorder in which a drooping eyelid obscures the pupil, hindering vision in severe cases. Ptosis is generally diagnosed by measuring eyelid morphological parameters, such as the levator muscle strength, lid fissure height, and limbal reflex distance, based on typical clinical symptoms. Surgical therapy is the main treatment for ptosis (Mahroo et al., 2014). Tabuchi et al. (2022) performed an automatic diagnosis of ptosis using a pre-trained MobileNetV2 CNNi applied to photos of patients taken with an iPad Mini. Hung et al. (2022) realized the automatic identification of monocular appearance photos of ptosis patients based on a VGG-16 neural network, and the results showed that AI outperformed GPs in diagnosing ptosis. Combined with devices such as smartphones, the analysis of eye appearance based on AI can be useful in further clinical scenarios. AI provides an objective tool for measuring eyelid morphological parameters and planning surgery strategies instead of relying on the experience of surgeons. Song et al. (2021b) developed a gradient-boosted decision tree (GBDT) for choosing ptosis surgery strategies and trained it with 3D models created by photographing and scanning the eyes of ptosis patients with a structured light camera. The AI model evaluates the external ocular photographs and the 3D model to determine whether surgery is required and establish the surgery strategy to follow. Lou et al. (2021) evaluated the outcome of ptosis surgery by comparing pre- and postoperative values of eyelid morphological parameters, such as MRD1 and MRD2, which were automatically measured by a UNet from ocular appearance photographs of the patients.

### Artificial intelligence diagnosis and prediction based on external ocular photographs

Oculoplastic surgery involves the aesthetic restoration and predicting postoperative outcomes through AI can help the surgeon develop a personalized plastic surgery plan. The major purpose of oculoplastic surgery is to realize the expected aesthetic goals. However, it is hard to judge the expected aesthetic results due to a variety of subjective factors (Swanson, 2011). Establishing an objective facial beauty standard is still controversial. Zhai et al. (2019) proposed a new facial detection method based on a transfer learning CNN, which has better classification accuracy than previous geometric assessment methods, laying a foundation for the prediction of oculoplastic surgery effects. Yixin et al. explored the effect of the eyelid on oculoplastic surgery and aesthetic outcomes by comparing the postoperative metrics of oculoplastic patients assessed by the CNN model with those assessed only artificially. The CNN assessment group had better postoperative extent, lower eyelid skin wrinkles, eyelid tear troughs, skin shine, and aesthetic scores than the control group, suggesting that CNN is a beneficial tool for evaluating oculoplastic surgery (Yixin et al., 2022).

Eyelid and periocular skin tumors seriously affect the health and aesthetics of patients (Silverman and Shinder, 2017). Early preliminary screening through external photography helps detect and monitor these tumors. Seeja and Suresh (2019) trained a UNet to automatically segment skin lesions and differentiate melanoma from benign skin lesions, achieving reliable results in the segmentation and diagnosis of melanoma. Li et al. (2022b) used a faster region-based CNN and a DL classification network to build an AI system that automatically detects malignant eyelid tumors from ocular external photographs, obtaining positive performance on both internal and external test sets (AUC ranging from 0.899 to 0.955). CNNs could fully mine image information and distinguish deep features from external photography to detect subtle eyelid and skin tumors that are elusive to the naked eye, thus helping reduce misdiagnosis and missed diagnosis.

Changes in ocular appearance, such as retraction of the upper eyelid, strabismus, and proptosis, are crucial in the diagnosis of TAO (Hodgson and Rajaii, 2020). Huang et al. (2022) used the ResNet-50 model to obtain an automatic diagnosis of TAO based on external ocular photographs. Karlin J et al. (2022) developed a DL model for detecting TAO based on external ocular photographs. A set comprising 1944 photographs from a clinical database was used for training, and a test set of 344 additional images was used to evaluate the trained DL network. The accuracy of the model on the test set was 0.892, and heatmaps showed that the model could identify pixels corresponding to the clinical features of TAO. Orbital decompression surgery can alleviate the symptoms of eye protrusion and repair the appearance of patients with TAO. According to the 2021-EUGOGO guidelines, orbital decompression surgery is the recommended treatment strategy for patients with severe TAO (Smith, 2021). Yoo et al. (2020) trained a conditional generative adversarial network (GAN) using pre- and postoperative external ocular photographs of patients with orbital decompression. The trained GAN could convert the preoperative external ocular photographs into predictive postoperative images, which were similar to the real postoperative condition, suggesting that GAN might be a new tool for the prediction of oculoplastic surgery results. Table 2 summarizes the aforementioned AI-related studies on external ocular images.

**TABLE 2:** AI-related studies utilizing external ocular images.

Authors	Study goals	Imaging data type	Dataset	Network model	Accuracy	AUC
Moriyama et al. (2006)	Eye motion tracking	External ocular images	—	Generative eye region model	—	—
Van Brummen et al. (2021)	Segmentation of regions such as iris and eyebrow	Photographs of periorbital areas	418 images	ResNet-50	—	—
Bahceci Simsek and Sirolu, (2021)	Evaluation of postoperative changes	Full-face photographs	55 patients	DLIBML toolkit	—	—
Chen et al. (2021)	Measurement of eyelid paraments	External ocular images	411 participants	MAIA software	—	—
Tabuchi et al. (2022)	Classification of images taken with a tablet device of patients with blepharoptosis diagnosis	Eyelid images	1,276 images	Pre-trained MobileNetV2 CNNi	0.828	0.900
Hung et al. (2022)	Identification of monocular appearance photos of ptosis patients	External ocular images	782 images	VGG-16	0.90	0.987
Song et al. (2021b)	Determination of the choices of ptosis surgery strategies	External ocular images	152 eyes	Gradient-boosted decision tree (GBDT)	0.826	0.795
Lou et al. (2021)	Evaluation of ptosis surgery outcome	External ocular images	103 patients (135 ptotic eyes)	U-Net (Attention R2U-Net)	—	—
Yixin et al. (2022)	Exploration of the effect of eyelid on oculoplastic surgery and aesthetic outcomes	External ocular images	64 patients	Multichannel CNN	0.988	—
Huang et al. (2022)	Diagnosis of TAO	Facial images	3,120 eyes	ResNet-50 U-Net	Eye location: 0.980. Cornea: 0.930. Sclera segmentation: 0.870	Over 0.850
Li et al. (2022b)	Automatic detection of malignant eyelid tumors	External ocular images	Development set (*n* = 1,258). External test set (n = 309)	Faster-RCNN	—	AUCs ranged from 0.899 to 0.955
Karlin J et al. (2022)	Detection of thyroid eye disease	External ocular images	Training set (*n* = 1994). Test set (*n* = 344)	ResNet-18	0.892	
Yoo et al. (2020)	Synthesis of realistic postoperative appearance for orbital decompression surgery	External ocular images	500 preoperative images and 500 postoperative images	Generative adversarial network (GAN)	—	0.957

## Artificial intelligence-based techniques using other image data types

Tear spillage is a major symptom of lacrimal duct obstruction (LDO), and its incidence in rural areas is gradually increasing (Brendler et al., 2013). The use of anterior segment optical coherence tomography (AS-OCT) to assess the tear meniscus is considered a more objective non-invasive diagnostic procedure. Imamura et al. used DenseNet-169 and pooled DL models (VGG-16, ResNet-50, DenseNet-121, DenseNet-169, Inception ResNet-V2, and Inception-V3) to detect patients with LDO from AS-OCT images. The trained network models exhibited remarkable reliability in marking the areas of the tear meniscus (Imamura et al., 2021).

Pathological examination is the gold standard for diagnosing the nature of ocular tumors. However, traditional pathological examination results are influenced by the experience of the physician, which takes a large amount of time from specimen submission to result confirmation (Heran et al., 2014). AI is not influenced by subjective factors and can process a large number of specimens in a short time. Wang et al. (2020b) used AI technology to automatically diagnose malignant melanoma of the eyelid from pathological sections. They also developed a random forest model to grade tumor malignancy, suggesting that AI may be a future tool for the rapid screening and grading of tumor pathology.

Jiang et al. (2022) proposed a DL framework for the automatic detection of malignant melanoma (MM) of the eyelid based on self-supervised learning (SSL). The framework consisted of a self-supervised model for detecting MM regions at the patch level and another model for classifying lesion types at the slide level. Considering that the differential diagnosis of basal cell and sebaceous carcinomas of the eyelid is highly dependent on the experience of the pathologist, Luo et al. (2022) proposed a fully automated differential diagnostic method based on whole slide images (WSIs) and DL classification, achieving an accuracy of 0.983 for the trained network model.

In addition, AI-decision models can be established based on various types of patient information. Song et al. (2022) trained an ML model using a database that contained both ocular surface characteristics and demographic information (gender, age) of patients with lacrimal sacculitis. Tan et al. (2017) established an alternating decision tree to predict the risk of reconstructive surgery after eyelid basal cell carcinoma (pBBC) resection which provides a new prediction model based on a database with various patient information.

## Discussion

The acquisition and analysis of imaging data are crucial in the treatment of orbital and eyelid diseases. In this paper, we discuss the advantages and limitations of AI technology for diagnosing orbital and eyelid diseases by analyzing the different characteristics of image data and the current problems and potential approaches to promote the development of AI-based technology in this field.

Orbital and eyelid diseases are primarily caused by inflammatory (Lutt et al., 2008), metabolic, and traumatic factors (Li et al., 2020). The anatomy integrity of the orbital and eyelid not only protects and supports important structures, such as the eyeball and optic nerve, but is also critical to the aesthetic appearance of the patient’s face (Huggins et al., 2017). AI converts traditional medical images into matrix data and supports clinical decision-making by developing models and analyzing the matrix data (Mintz and Brodie, 2019). Structural segmentation of orbital CT/MRI images using AI might assist in endoscopic and 3D-print surgery and lay a foundation for robotic surgery (Wang et al., 2022). In addition, the automatic measurement of eyelid morphological parameters based on external ocular photographs provides a new tool for developing individualized eyelid surgical strategies (Bahceci Simsek and Sirolu, 2021). Thus, AI technology for diagnosing and treating orbital and eyelid diseases, which remains in its infancy, has great potential for broad clinical application.

Imaging data play an important role in the diagnosis and treatment of orbital and eyelid diseases, providing an adequate source of data for AI training. Non-invasive Orbital CT examination is easy and fast to perform (Lee et al., 2004). Orbital MRI examination is free of ionizing radiation damage and is superior in revealing soft tissue. Compared with MRI examinations, CT images are noisier (Hamwood et al., 2021). Therefore, the traditional UNet algorithm is better suited to training with CT images because it extracts rich feature scales and can effectively filter local noise (Pan et al., 2022). External ocular photographs serve as a unique type of imaging data for orbital and eyelid diseases and provide information for eyelid surgery decisions. Compared with other types of medical images, external ocular photographs are non-invasive and can be easily taken by doctors and patients with smartphones, which breaks the barrier of expensive image equipment and facilitates the application of AI (Chen et al., 2021). Furthermore, automatic facial recognition and eye-tracking technology, which have been widely used in safety inspection, instrument development, etc., could also be applied to AI research based on external ocular photographs (Asaad et al., 2020). In addition, visual field tests, OCT, and CT of the optic-nerve canal also play an active role in the diagnosis of orbital and eyelid diseases. The multimodal diagnostic images provide adequate raw datasets for training AI models and validating their performance.

Although AI analysis of imaging data of orbital and eyelid diseases has, there are some limitations in its development (Mintz and Brodie, 2019; Yang et al., 2021). Uneven disease prevalence and small sample sizes for certain rare diseases cause oversampling when training AI models for specific types of diseases, resulting in poor generalization and a lack of adaptability to new data. Several studies have shown that the category imbalance problem can be solved by weighting the data differently when computing the loss function (Liu et al., 2021; Luo et al., 2021). Moreover, the current AI datasets of orbital and eyelid diseases are generally obtained from the same medical institution. However, it is difficult to obtain standardized data because of the differences in the examination equipment used by different medical institutions. Image-based AI research requires large sets of standard, annotated imaging data, which are still scarce in the case of orbital and eyelid diseases as compared, for instance, with ImageNet. Transfer learning may offer a good solution to the lack of imaging data. When obtaining a large dataset or labeling the data is difficult, learning can be transferred from a task with sufficient data that is easily labeled and similar to the target task. Liu et al. (2020) modified ResNet-152, which was pre-trained on ImageNet, through transfer learning to classify left and right optic discs with an accuracy of 0.988, thus demonstrating a new solution to the lack of data in orbital and eyelid diseases. In addition, we can increase the amount of data through data augmentation by rotating, panning, zooming, or changing the brightness or contrast of the images. For example, Song et al. (2021a) performed 200 rotations on the training data for a CNN to increase the dataset size and reduce overfitting. When verified, the overfitting of the trained CNN remarkably decreased. There are also some drawbacks regarding the quality of imaging data in orbital and eyelid diseases, which have limited the development of AI-related research. For example, it is difficult to obtain standardized orbital CT/MRI images due to the long scanning time, different equipment, and variable experience of the operators. Zhai et al. (2021) developed a method based on a signed distance field for the automatic calibration and quantitative error evaluation when processing orbital CT images, which provides a new tool to standardize CT/MRI images. Moreover, lighting variations prevent high-quality standardized external ocular photography. To address this problem, some studies have attempted to model the illumination templates and establish illumination-invariant algorithms (Li et al., 2007; Lu et al., 2017), whose main purpose is to make shapes and textures independent of illumination variations. Lastly, ethical considerations and patient privacy issues associated with external ocular photography also require in-depth deliberation.

Overall, although there are still some limitations to the advance of AI-based research on orbital and eyelid diseases, as large databases are established and shared and as new neural networks that more closely resemble biological neurons are developed, further development of such AI applications is expected to occur, leading to the next breakthrough in ophthalmology.

## Conclusion

AI technology has a significant potential for application in the automatic diagnosis and precise quantification of orbital and eyelid diseases. AI is more objective than manual methods, can process large amounts of data in a short time, and, thus, could assist physicians in clinical decision-making and surgical design. The predictive capabilities of AI may also play an active role in assessing the outcome of oculoplastic surgery. As computer algorithms are updated and high-quality datasets become available, AI will play a broader role in the assessment of orbital and eyelid disorders in the future.
